# Dosimetric comparison of TomoDirect, helical tomotherapy, and volumetric modulated arc therapy for postmastectomy treatment

**DOI:** 10.1002/acm2.12989

**Published:** 2020-07-27

**Authors:** Wannapha Nobnop, Panchalee Phakoetsuk, Imjai Chitapanarux, Damrongsak Tippanya, Darat Khamchompoo

**Affiliations:** ^1^ Division of Radiation Oncology Faculty of Medicine Chiang Mai University Chiang Mai Thailand; ^2^ Northern Thai Research Group of Radiation Oncology (NTRG‐RO) Faculty of Medicine Chiang Mai University Chiang Mai Thailand; ^3^ Radiation Therapy Center Nakornping Hospital Chiang Mai Thailand; ^4^ Faculty of Medicine Chiang Mai Cancer Registry Maharaj Nakorn Chiang Mai Hospital Chiang Mai University Chiang Mai Thailand

**Keywords:** helical tomotherapy, IMRT, postmastectomy, VMAT, TomoDirect

## Abstract

**Purpose:**

To compare dose to the targets and organs at risk (OARs) in different situations for postmastectomy patients who require radiation to the chest wall with or without regional nodal irradiation when using three treatment techniques.

**Methods and materials:**

Thirty postmastectomy radiotherapy (PMRT) patients previously treated by helical tomotherapy (HT) at our institution were identified for the study. The treatment targets were classified in three situations which consisted of, the chest wall (CW) only, the chest wall plus supraclavicular lymph nodes (CW + SPC), and the chest wall plus supraclavicular and whole axillary lymph nodes irradiation (CW + SPC+AXLN). The volumetric modulated arc therapy (VMAT) plans and Tomodirect (TD) plans were created for each patient and compared with HT treatment plans which had been treated. The target coverage, dose homogeneity index (HI), conformity index (CI), and dose to OARs were analyzed. The quality scores were used to evaluate the appropriate technique for each situation from multiparameter results.

**Results:**

The HT and VMAT plans showed the advantage of target coverage and OARs sparing for the chest wall with regional nodal irradiation with the higher plan quality scores when compared with TD plans. However, TD plans demonstrated superiority to contralateral breast sparing for the chest wall without regional nodal situation reaching the highest of planned quality scores. HT plans showed better HI, CI, and target coverage (*P* < 0.01) than TD and VMAT plans for all patient situations. Volumetric modulated arc therapy plans generated better contralateral breast and heart sparing at a lower dose than HT.

**Conclusion:**

The arc‐based techniques, HT and VMAT plans, provided an advantage for complex targets in terms of target coverage and OARs sparing. However, the static beam TD plan was superior for contralateral organ sparing meanwhile achieving good target coverage for the chest wall without regional node situations.

## 1 INTRODUCTION

Postmastectomy radiotherapy (PMRT) presents a complex target volume, generally consisting of the chest wall (CW) and regional lymph nodes. The challenge of treatment planning is that it covers a large, superficial surface which is a thin area and a concave‐shaped target.^1^ In our clinic, postmastectomy patients were classified in three situations, chest wall only irradiation, chest wall plus supraclavicular nodes irradiation and chest wall including supraclavicular and axillary node irradiation. Each situation presents a variety of target complexity, which affects the selection of treatment techniques for the radiation oncologist.

Previously, PMRT in our clinic was treated with a mixed‐beam technique consisting of three‐dimensional (3D) technique with medial and lateral tangential field for CW. For increasing the skin dose, 1.0 cm. bolus used for half of the treatment course. Two 3D plans to be generated, the bolus and the nonbolus fractions. The anterior x‐ray field was used for supraclavicular lymph nodes (SPC) with prescribed point at 3–4 cm. depth and posterior x‐ray field prescribed point at midline depth was used for axillary nodes combined with anterior electrons to treat internal mammary nodes. Subsequently, helical tomotherapy (HT) often becomes the treatment of choice for PMRT due to improved conformality to the target, while sparing the OARs.^2^

Tomotherapy can be performed in two modes. First is the HT delivery mode, a technique to treat continuous gantry rotations around the patient, using thousands of narrow beamlets, which are individually optimized to the target. However, TomoDirect (TD) is a nonrotational treatment by coplanar static beams, with the couch moving at a constant speed through a fixed binary multileaf collimator (MLC) that modulates the beam. After the patient has been treated with one gantry angle, the gantry is rotated to a different angle and the patient is again passed through the bore for the delivery of subsequent fields.^3^

Heretofore, volumetric modulated arc therapy (VMAT) intensity‐modulated delivery technique available with a linear accelerator launched in our center. Volumetric modulated arc therapy is a continuous modulation of the MLC, dose rate, and variable gantry speed to deliver highly conformal dose distributions in a short period of time.^4^ Volumetric modulated arc therapy has become another choice of PMRT treatment in our clinic. Therefore, the objectives of this study were to compare the dose to the target and organs at risk (OARs) in different situations of left‐sided PMRT patients requiring radiation to the chest wall with or without regional nodal irradiation when using the three treatment techniques, TomoDirect, Helical tomotherapy, and VMAT. Then we evaluated which advantages of each technique were suitable for each situation in our institute.

## 2 MATERIALS AND METHODS

### 2.A. Patients

**Fig. 1:**
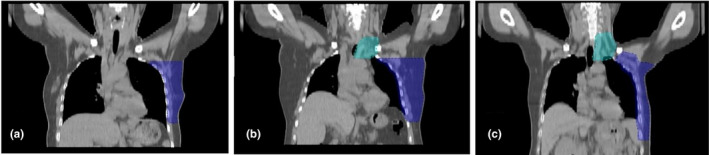
The coronal view of the computed tomography (CT) images and structure for the patients (a) the first situation (chest wall only), (b) the second situation (chest wall included supraclavicular lymph nodes), and (c) the third situation (chest wall included supraclavicular and axillary lymph nodes irradiation).

This study included 30 patients who were treated by helical tomotherapy at our institution for left‐sided PMRT between January 2017 and December 2018. The treatment targets were classified in three situations, with ten patients per each situation. Figure 1 illustrated the CT images and structure for the patients in the first situation received only chest wall (CW) treatment. The second situation was the patients with chest wall treatment that included supraclavicular lymph nodes (CW + SPC). The third situation treated the chest wall including supraclavicular and whole axillary lymph nodes irradiation (CW + SPC + AXLN). All patients underwent 3D simulation in the supine position on the wing board (CIVCO, USA) with both arms up above the head. Computed tomography (CT) as performed with a slice thickness of 3 mm. and using radiopaque wires to define the scars and field borders on the patients’ skin during CT simulation.

### 2.B. Dose prescription and dose constraint

The target volume of the chest wall and regional nodes were localized separately. The prescription dose for all patients was 50 Gy in 25 fractions. The dosimetric constraints were determined from various publications and recommendations.^5^, ^6^, ^7^, ^8^, ^9^, ^10^, ^11^, ^12^ The dose to OARs was divided into two dose constraints (Table 1), the patients in the first situation followed the constraint for the CW only irradiation. The second constraint, CW plus regional nodes determined for the patients receiving treatment of the CW and regional nodes which were the second and the third situations. Because only the left‐side PMRT patients were enrolled in this study, the dosimetric doses for heart, left anterior descending artery (LAD) were compared in each treatment technique.

**Table 1:** The dose constraints for postmastectomy radiotherapy (PMRT) patients with irradiated planning target volume (PTV) chest wall (CW) and chest wall plus regional nodes (CW + RN).

Structure	Chest wall only irradiation		Chest wall + regional nodes irradiation	
	Dose (Gy)	Volume (%)	Dose (Gy)	Volume (%)
PTV (CW, CW + RN)	<53.5	2	<53.5	2
	50	50	50	50
	>47.5	95	>47.5	95
Ipsilateral lung	20	20	20	30
			30	20
Contralateral lung	5	10	5	20
Contralateral breast	5	10	5	10
	8	2	8	2
Heart	20	15	20	15
	10	20	10	20
Heart (mean dose)	8		8	
Spinal cord	20	2	20	2
Left anterior descending artery (LAD)	50	2	50	2
Esophagus	52.5	2	52.5	2

### 2.C. Treatment planning

This was a retrospective study with images from CT simulation with structure delineation for 30 PMRT patients imported into two treatment planning systems. All patients underwent 3D simulation in the supine position on the wing board (CIVCO, USA) with both arms up above the head. Multislice CT simulation (Somatom; Siemens, Germany) as performed with a slice thickness of 5 mm. and using radiopaque wires to define the scars and field borders on the patients' skin during CT simulation. The TomoTherapy treatment planning system, a planning station version 5.1.1.6 (Accuray, Incorporated, Sunnyvale, CA, USA) was used to create the TomoDirect and Helical tomotherapy plans. However, VMAT plans were created using Monaco version 5.11.02 (Elekta AB, Stockholm, Sweden). All 30 PMRT patients were planned in three treatment techniques, to decrease the bias of the treatment planners. The three medical physicists specific in each technique were assigned with blinding to the results from other techniques. Therefore, there were a total of 90 treatment plans for the dosimetric comparison.

#### 2.C.1. TomoDirect plan setting

All plans used a jaw width of 2.5 cm, a pitch of 0.25, and a modulation factor between 3.0 and 3.2. The beam placement for chest walls used seven beams in IMRT mode, three beams for medial tangential, and three beams for the lateral tangential direction. We added another beam in anterior oblique direction for improved target conformity. TomoDirect mode skin flash was applied to compensate for the intrafraction movement by retracting three leaves (1.8 cm). In the case of the second and the third situations of patient who were treated for SPC and full axillary lymph nodes, the beams were placed to the planning target volume of regional nodes (PTV‐RN) with three directions in anterior and two oblique beams.

#### 2.C.2. Helical tomotherapy plan setting

HT treatment plans were created using a jaw width of 2.5 cm, a pitch of 0.43, and a modulation factor of 3.0. We created a directional block to limit the entrance dose to OARs for both lung, contralateral breast, heart, and spinal cord. The optimization iterations were completed when the planning goals were met or until the plan could no longer be improved.

#### 2.C.3. Volumetric modulated arc therapy plan setting

VMAT treatment plans were created using two partial VMAT arcs of 210°–240° with start and stop angles of the first arc set to 295° and 145°, respectively, for chest wall only irradiation and 275° and 155°, respectively, for chest wall with regional nodes irradiation. The skin flash function was applied to compensate for the intrafraction movement by retracting four leaves (2.0 cm).

### 2.D. Dosimetric comparison metrics

The targets of each situation were compared in three treatment techniques from the following quantities: target coverage (V_95%_), homogeneity index (HI), and conformity index (CI), calculation:^13^, ^14^

$$\text{HI}=\frac{D_{2\%}‐D_{98\%}}{D_{50\%}}$$

where $D_{2\%},D_{98\%,}$ and $D_{50\%}$ denote the near‐minimum, near‐maximum and median dose, respectively. An HI of zero indicates that the dose distribution is almost homogeneous. The conformity index, calculated as^1^

$$\text{CI}=\frac{TV_{PIV_{95\%}}}{\mathrm{TV}}x\frac{TV_{PIV_{95\%}}}{PIV_{95\%}}$$

where TV is the target volume, PIV is the volume of the 95% of prescribed isodose value and $TV_{\mathrm{PIV}}$ is the volume of the target that is covered by the 95% of the prescribed isodose value. The larger value of CI representing better dose conformity. To better analyze the most superior technique for each situation from the multiparameter results, we summarize from the quality score table of the plans.^13^ In the quality score table, point 1 means that technique showed significant superior (*P* < 0.05) when compared with another technique among the different plans, otherwise scored to 0. The best index could get 1 point in each technique. Dose to OARs was evaluated using a dosimetric comparison between the three techniques. The one‐way analysis of variance (ANOVA) test and the paired sample t‐test were used on each technique of comparison metrics to determine the statistical significance, with a threshold of *P* < 0.05; SPSS statistical software version 17 for statistical analysis. This study was approved by the Institutional Review Board of the Faculty of Medicine Chiang Mai University (study code RAD‐2560‐04997/Research ID: RAD‐2560‐04997).

## 3 RESULTS

### 3.A Planning target volume dose comparison

**Fig. 2:**
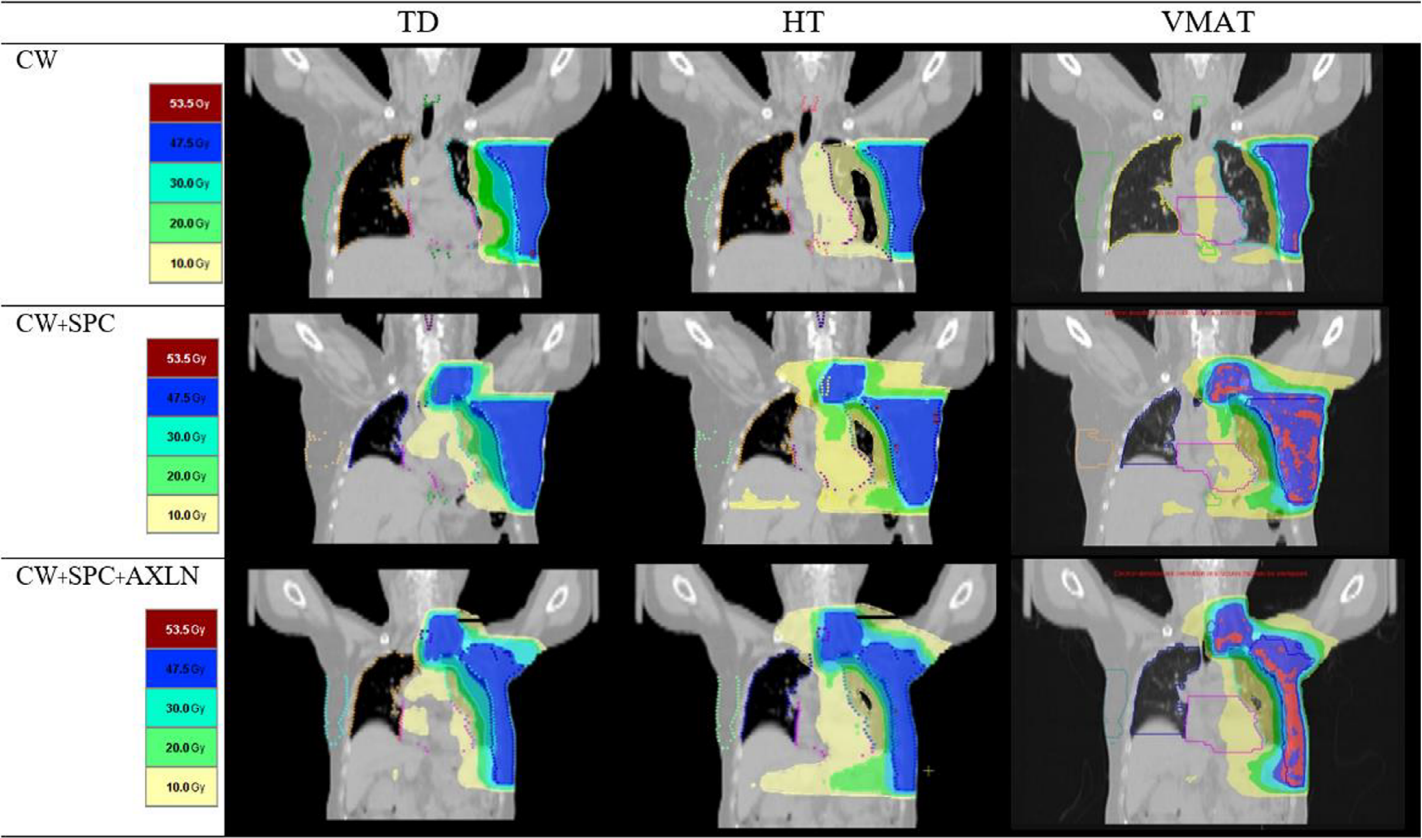
The dose distribution of coronal plane by TomoDirect (TD), Helical tomotherapy (HT) and Volumetric modulated arc therapy (VMAT) plans for three situations of patient (a) chest wall only; CW, (b) chest wall + supraclavicular lymph nodes; CW + SPC and (c) chest wall + supraclavicular nodes + axillary nodes; CW + SPC + AXLN irradiation.

Figure 2 shows the dose distribution comparison of TD, HT, and VMAT plan in the coronal planes in three situations of patients. The HT plan illustrated the smallest volume of the hot spot area for 107% of the prescription dose when compares with TD and VMAT for all three situations, indicating better homogeneity with HT for both chest wall and chest wall plus regional nodes irradiation as shown in Table 2.

**Table 2:** Mean and standard deviations of dose parameters in three treatment techniques.

Variables	TD Mean (SD)	HT Mean (SD)	VMAT Mean (SD)	*P*‐value
CW only				
‐PTV CW				
V_95%_	95.3 (0.7)	98.8 (0.6)	95.5 (1.6)	<0.01
HI	0.153 (0.015)	0.089 (0.009)	0.142 (0.012)	<0.01
CI	0.709 (0.122)	0.808 (0.079)	0.768 (0.119)	<0.01
CW + SPC				
‐PTV CW				
V_95%_	95.7 (0.8)	97.4 (0.8)	95.6 (0.7)	<0.01
HI	0.149 (0.012)	0.101 (0.020)	0.174 (0.024)	<0.01
CI	0.657 (0.130)	0.816 (0.089)	0.788 (0.112)	<0.01
‐PTV RNI				
V_95%_	98.8 (0.7)	99.8 (0.2)	98.6 (1.4)	<0.01
HI	0.085 (0.021)	0.071 (0.011)	0.152 (0.065)	<0.01
CW + SPC + AXLN				
‐PTV CW				
V_95%_	95.7 (0.4)	97.1 (0.9)	95.3 (0.5)	<0.01
HI	0.144 (0.014)	0.112 (0.012)	0.176 (0.023)	<0.01
CI	0.683 (0.189)	0.790 (0.101)	0.761 (0.106)	<0.01
‐PTV RNI				
V_95%_	98.9	99.7	98.1	<0.01
HI	0.088 (0.019)	0.063 (0.011)	0.112 (0.027)	<0.01

Abbreviations: AXLN, axillary nodes; CI, conformity index; CW, chest wall; HI, homogeneity index; HT, TomoHelical; PTV, planning target volume; RNI, regional nodes; SPC, supraclavicular lymph nodes; TD, TomoDirect; VMAT, volumetric modulated arc therapy.

The HT plans showed to be superior in target coverage, HI and CI for all situations of PTV chest wall and PTV regional node as shown in Table 2. For PTV chest wall, the average of target coverage (V_95%_) for HT was 98.8%, 97.4%, and 97.1% for CW, CW + SPC, and CW + SPC + AXLN, respectively. Moreover, HI and CI were significantly better in HT plans by HI = 0.089, 0.101, and 0.112 for CW, CW + SPC, and CW + SPC + AXLN, respectively. The CI for HT were 0.808, 0.816, and 0.790 for CW, CW + SPC, and CW + SPC + AXLN, respectively. The HT plans also explored the superior for target coverage, HI and CI for PTV regional node CW + SPC and CW + SPC + AXLN.

### 3.B. Organs at risk (OARs) dose comparison

#### 3.B.1. Chest wall without nodal irradiation

Table 3 shows the TD plan was significantly lower than the other two plans for contralateral breast, spinal, and esophagus sparing of chest wall without regional nodal situation and reached the highest score of plan quality as shown in Table 4.

**Table 3:** Mean and standard deviation of dose parameters for the organs at risk.

Structure	Metric	TD Mean (SD)	HT Mean (SD)	VMAT Mean (SD)	*P*‐value
CW only					
Ipsilateral lung	V_20Gy_	27.0 (6.3)	20.2 (1.2)	19.4 (1.4)	0.04
Contralateral lung	V_5Gy_	8.6 (3.6)	8.0 (1.8)	8.0 (3.3)	0.01
Contralateral breast	V_5Gy_	2.4 (1.5)	8.8 (1.8)	8.5 (2.6)	<0.01
	D_2%_	6.9 (3.6)	7.1 (0.5)	7.8 (1.5)	0.27
Heart	V_20Gy_	19.8 (8.7)	14.0 (2.3)	12.9 (1.5)	0.06
	V_10Gy_	41.5 (7.0)	70.9 (6.0)	39.8 (8.4)	<0.01
	D_mean_	12.7 (2.8)	14.2 (0.7)	11.2 (0.9)	<0.01
LAD	D_max_	44.6 (3.4)	43.3 (4.5)	44.5 (3.2)	0.51
Spinal cord	D_max_	6.0 (4.0)	13.0 (1.4)	8.9 (2.9)	<0.01
Esophagus	D_max_	15.0 (20.1)	18.8 (3.1)	15.2 (2.4)	<0.01
CW + SPC					
Ipsilateral lung	V_20Gy_	37.0 (5.7)	26.9 (1.9)	36.7 (4.0)	<0.01
	V_30Gy_	26.9 (5.1)	18.3 (1.4)	20.0 (1.6)	<0.01
Contralateral lung	V_5Gy_	8.9 (3.3)	14.8 (3.0)	17.4 (2.3)	<0.01
Contralateral breast	V_5Gy_	5.9 (4.0)	11.6 (2.5)	8.8 (1.0)	<0.01
	D_2%_	11.0 (8.2)	7.4 (0.6)	6.1 (0.2)	0.02
Heart	V_20Gy_	25.5 (8.8)	20.5 (9.8)	15.2 (3.6)	<0.01
	V_10Gy_	45.7 (12.4)	71.5 (10.8)	60.1 (6.9)	<0.01
	D_mean_	15.1 (2.0)	15.8 (1.1)	13.5 (1.1)	<0.01
LAD	D_max_	45.4 (12.3)	49.5 (1.6)	47.0 (1.2)	<0.01
Spinal cord	D_max_	11.2 (2.1)	25.1 (4.7)	16.6 (0.8)	<0.01
Esophagus	D_max_	50.9 (1.4)	50.7 (0.6)	49.1 (0.9)	0.02
CW + SPC + AXLN					
Ipsilateral lung	V_20Gy_	38.3 (4.7)	28.1 (1.7)	37.3 (3.5)	<0.01
	V_30Gy_	27.8 (2.7)	19.3 (1.0)	20.2 (1.6)	<0.01
Contralateral lung	V_5Gy_	14.9 (5.4)	15.9 (1.6)	15.6 (5.1)	0.74
Contralateral breast	V_5Gy_	8.8 (6.4)	12.3 (1.7)	10.8 (5.0)	<0.01
	D_2%_	11.3 (7.6)	7.5 (0.6)	6.2 (0.6)	<0.01
Heart	V_20Gy_	25.7 (9.7)	16.8 (2.7)	15.2 (2.2)	0.01
	V_10Gy_	54.9 (7.0)	75.5 (5.4)	62.6 (5.8)	<0.01
	D_mean_	15.3 (2.3)	16.0 (0.7)	13.6 (1.0)	<0.01
LAD	D_max_	47.9 (2.9)	50.1(1.5)	45.1 (4.6)	<0.01
Spinal cord	D_max_	9.8 (2.3)	21.7 (2.8)	15.7 (1.0)	<0.01
Esophagus	D_max_	49.3 (2.7)	50.0 (1.3)	47.4 (3.3)	<0.01

Abbreviations: AXLN, axillary nodes; CI, conformity index; CW, chest wall; HI, homogeneity index; HT, TomoHelical; LAD, left anterior descending artery; SPC, supraclavicular lymph nodes; TD, TomoDirect; VMAT, volumetric modulated arc therapy.

**Table 4:** Planned score table of the three treatment techniques for three situations.

Structure	Metric	TD Mean (SD)	HT Mean (SD)	VMAT Mean (SD)
CW only				
PTV CW	V_95%_	0	1	0
	HI	0	1	0
	CI	0	1	0
Ipsilateral lung	V_20Gy_	0	1	1
Contralateral lung	V_5Gy_	0	0	0
Contralateral breast	V_5Gy_	1	0	0
	D_2%_	0	0	0
Heart	V_20Gy_	0	0	0
	V_10Gy_	1	0	1
	D_mean_	1	0	1
LAD	D_max_	0	0	0
Spinal cord	D_max_	1	0	1
Esophagus	D_max_	1	0	0
Total score		5	4	4
CW + SPC				
PTV CW	V_95%_	0	1	0
	HI	0	1	0
	CI	0	1	0
PTV RNI	V_95%_	0	1	0
	HI	0	1	0
Ipsilateral lung	V_20Gy_	0	1	0
	V_30Gy_	0	1	1
Contralateral lung	V_5Gy_	1	0	0
Contralateral breast	V_5Gy_	1	0	1
	D_2%_	0	0	1
Heart	V_20Gy_	0	1	1
	V_10Gy_	1	0	0
	D_mean_	0	0	1
LAD	D_max_	1	0	1
Spinal cord	D_max_	1	0	0
Esophagus	D_max_	0	0	1
Total score		5	8	7
CW + SPC + AXLN				
PTV CW	V_95%_	0	1	0
	HI	0	1	0
	CI	0	1	0
PTV RNI	V_95%_	0	1	0
	HI	0	1	0
Ipsilateral lung	V_20Gy_	0	1	0
	V_30Gy_	0	1	1
Contralateral lung	V_5Gy_	0	0	0
Contralateral breast	V_5Gy_	1	0	1
	D_2%_	0	0	1
Heart	V_20Gy_	0	1	1
	V_10Gy_	1	0	1
	D_mean_	0	0	1
LAD	D_max_	1	0	1
Spinal cord	D_max_	1	0	1
Esophagus	D_max_	0	0	1
Total score		4	8	9

Abbreviations: AXLN, axillary nodes; CI, conformity index; CW, chest wall; HI, homogeneity index; HT, TomoHelical; LAD, left anterior descending artery; PTV, planning target volume; RNI, regional nodes; SPC, supraclavicular lymph nodes; TD, TomoDirect; VMAT, volumetric modulated arc therapy.

#### 3.B.2. Chest wall with nodal irradiation

HT plans demonstrated to be significantly lower than the other two plans for ipsilateral lung sparing. However, VMAT showed the lowest dose to the heart and TD plans still showed a significantly lower dose for contralateral lung, breast, and spinal cord for all situations of patient treatments. The score of plan quality shows the advantage of the arc‐based IMRT over the TD plans for the complex target situation as shown in Table 4.

## 4 DISCUSSION

Dosimetric comparisons of different IMRT plans, static and arc‐based IMRT techniques for PMRT have been assessed in this study for evaluating the appropriate technique for each patient’s situation from multiparameter results. Regarding the chest wall only irradiation, TD showed advantages on multiple indices for almost all patients, contralateral lung, spinal cord, heart, etc. Zhao et al.^13^ reported that two‐field IMRT showed superior dosimetric parameters than VMAT. However, for the complex target, chest wall plus regional nodal, the arc‐based technique as HT and VMAT plan provided better dosimetry over than TD in terms of target coverage and normal tissue sparing.

Helical tomotherapy plans showed significantly better dose homogeneity and conformity. On the other hand, VMAT plans illustrated significantly better sparing of normal tissues, which is consistent with a previous study from Nichols et al.^1^ Regarding the plan quality score, TD plans demonstrated to be superior for the chest wall without regional nodal situation and reached the highest of plan quality scores when compared to HT and VMAT. However, arc‐based delivery showed the advantage of target coverage and OARs sparing for the chest wall with regional nodal irradiation with the higher plan quality scores when compared with TD plans. While the HT and VMAT plans showed to be comparable for all target situations.

**Fig. 3:**
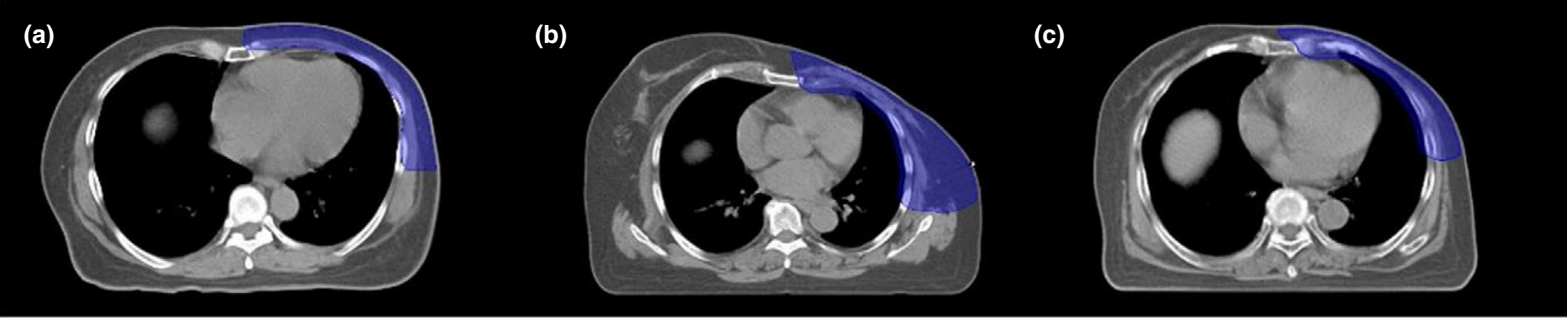
The transverse view of the computed tomography images of planning target volume chest wall structure (blue color) for one patient for each situation (a) the first situation (chest wall only), (b) the second situation (chest wall included supraclavicular lymph nodes), and (c) the third situation (chest wall included supraclavicular and axillary lymph nodes irradiation).

Regarding the heart dose, all treatment techniques could not pass the criteria for mean heart dose and V_10Gy_ because the PTV CW showed a very thin shape and close to the heart volume as shown in Fig. 3. The higher mean contralateral lung dose for TD plans due to the wide PTV and the body shape of patients. Other reasons were the beam direction of TD plans, six tangential direction, and another beam in anterior oblique direction may cause of increasing the low dose for contralateral lung.

For the beam on time comparison, Nichols et al.^1^ showed the superior of VMAT plans over the HT plan by effectively reducing the MUs and treatment time, which is consistent with this study. The VMAT plans showed the superior than TD and HT plans for all situations with significant different. The average beam on time of TD, HT, and VMAT plan were 819.7 (±78.8), 810.2 (±66.9), and 238.5 (±23.7) s, respectively. Bajali et al.^15^ reported the IMRT and VMAT plans could help to reduce dose the heart and ipsilateral lung while improving the PTV coverage, conformity, and homogeneity when compare with the conventional 3D plans. However, these techniques demonstrated to increase the volume of OAR receiving a low dose and required higher monitors unit (MU). To improve methods are needed for PMRT irradiation for reducing the low dose volume. The concept of hybrid IMRT that combines conventional fields with IMRT field for the optimal dose mixture were explored.^15^, ^16^

So, from the overall results and plan quality score we suggest choosing TD with the highest score which was suitable for chest wall only irradiation of PMRT. However, the VMAT and HT were the most suitable for chest wall plus regional node irradiation with the highest plan quality score.

## 5 CONCLUSION

The arc‐based techniques as HT and VMAT plans provided the advantage for complex targets in terms of target coverage and OARs sparing. However, static beam as TD plans showed to be superior for contralateral organ sparing meanwhile achieved the good target coverage for chest wall without regional node situation.

## CONFLICT OF INTEREST

No conflict of interest.
